# Biochemical characterization and gene structure analysis of the 24‐kDa glutathione transferase sigma from *Taenia solium*

**DOI:** 10.1002/2211-5463.13795

**Published:** 2024-03-21

**Authors:** Ricardo Miranda‐Blancas, Oscar Rodríguez‐Lima, Ponciano García‐Gutiérrez, Roberto Flores‐López, Lucía Jiménez, Rafael A. Zubillaga, Enrique Rudiño‐Piñera, Abraham Landa

**Affiliations:** ^1^ Departamento de Microbiología y Parasitología, Facultad de Medicina Universidad Nacional Autónoma de México Mexico; ^2^ Departamento de Química Universidad Autónoma Metropolitana‐Iztapalapa Mexico City Mexico; ^3^ Posgrado en Ciencias Biológicas Unidad de Posgrado Universidad Nacional Autónoma de México Mexico; ^4^ Departamento de Medicina Molecular y Bioprocesos, Instituto de Biotecnología Universidad Nacional Autónoma de México Cuernavaca Mexico

**Keywords:** cestode, enzyme stability, glutathione transferase, prostaglandin D_2_, sigma class, *Taenia*

## Abstract

*Taenia solium* can cause human taeniasis and/or cysticercosis. The latter can in some instances cause human neurocysticercosis which is considered a priority in disease‐control strategies and the prevention of mental health problems. Glutathione transferases are crucial for the establishment and long‐term survival of *T. solium*; therefore, we structurally analyzed the 24‐kDa glutathione transferase gene (*Ts24gst*) of *T. solium* and biochemically characterized its product. The gene promoter showed potential binding sites for transcription factors and xenobiotic regulatory elements. The gene consists of a transcription start site, four exons split by three introns, and a polyadenylation site. The gene architecture is conserved in cestodes. Recombinant Ts24GST (rTs24GST) was active and dimeric. Anti‐rTs24GST serum showed slight cross‐reactivity with human sigma‐class GST. A 3D model of Ts24GST enabled identification of putative residues involved in interactions of the G‐site with GSH and of the H‐site with CDNB and prostaglandin D_2_. Furthermore, rTs24GST showed optimal activity at 45 °C and pH 9, as well as high structural stability in a wide range of temperatures and pHs. These results contribute to the better understanding of this parasite and the efforts directed to fight taeniasis/cysticercosis.

## Graphical abstract

Glutathione transferases (GST) are involved in detoxification and immunomodulation, allowing the establishment and development of cestodes in the host. *Taenia solium* has a 24‐kDa GST sigma class inducible gene that encodes a stable enzyme in a wide range of pHs and temperatures. Its enzymatic activity differs from other GSTs in the parasite, suggesting a different role to detoxification.

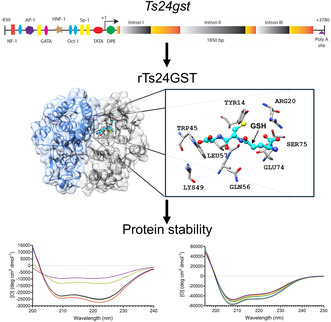

**Abbreviations**
Ahr: aryl hydrocarbon receptor
Anti‐rTs24GST: antibody for rTs24GST
ARNT: AhR nuclear translocator
CD: circular dichroism
CDNB: 1‐chloro‐2,4‐dinitrobenzene
DPE: downstream promoter element
ECA: ethacrynic acid
GSH: glutathione
GST: glutathione transferase
HS: 2‐hydroxyl disulphide
NBC: 4‐nitrobenzyl chloride
NCC: neurocysticercosis
PD: D‐prostanoid
PGD: prostaglandin
rTs24GST: recombinant Ts24GST
TBP: TATA binding protein
*Ts24gst*
: 24‐kDa glutathione transferase gen
Ts24GST: 24‐kDa glutathione transferase sigma class from *Taenia solium*
TSS: transcription start site
XRE: xenobiotic response elements

*Taenia solium* causes human taeniasis/cysticercosis. In the small intestine, the adult stage causes taeniasis that is practically asymptomatic, while the larval stage causes cysticercosis in humans and pigs [1]. Human neurocysticercosis (NCC) is a major foodborne parasitosis and is considered a priority for the establishment of disease‐control strategies and the prevention of mental health problems. Infection causes several neurological symptoms and 30% of epilepsy in countries where it is endemic [2, 3, 4, 5, 6, 7]. In addition, NCC leads to high costs to health systems. In Mexico, the annual cost of care per patient with NCC associated with epilepsy and severe chronic seizures was estimated between US$288 and US$1313 in 2015 [8].

Glutathione transferases (GSTs, EC 2.5.1.18) play a crucial role in the establishment and long survival of the helminth *T. solium* in its host and in its immunomodulatory action. They are also important for the transport of molecules and catabolism of aromatic amino acids that are related to the pathogeny of the disease. Thus, they are candidate targets for drugs, vaccines, and therapies [9, 10, 11, 12]. For example, schistosome glutathione transferase P28GST prevents intestinal inflammation in experimental colitis through a Th2‐type response with mucosal eosinophils [13].

Glutathione transferases are divided into three large groups: cytosolic (cGST), mitochondrial, and microsomal GSTs [14, 15]. In cestodes, only microsomal GSTs and cGST have been characterized, and their principal role seems to be detoxification. The cGST catalyzes the conjugation of reduced glutathione (GSH) to various xenobiotics and is active as a dimer with subunits of 20–30 kDa [16, 17].

Cytosolic glutathione transferases present two binding sites per monomer: one for glutathione binding (G‐site) and the other for xenobiotic binding (H‐site), and both form active sites of the enzyme. A third site is located at the dimer interface and is called the ligandin site (L site), which transports non‐substrates for the enzyme, such as bilirubin, heme, steroids, hormones, drugs, and bile salts [18, 19, 20, 21]. In *T. solium*, there are four cGSTs that have been described and classified by their substrates, inhibitors, kinetical properties, and protein structure. These four cGSTs belong to alpha‐mu (Ts26GST), mu (Ts25GST), sigma (Ts24GST = TsMαGST), and omega (Ts27GSTO, unpublished results) classes [22, 23, 24]. Recently, an omega class GST named EgrGSTO was reported in *Echinococcus granulosus* [25]. In cestodes, a sigma‐class GST has been associated with detoxification of H_2_O_2_, drug resistance, and an immunomodulation process, of which the latter is due to the property of prostaglandin D_2_ synthase [26, 27].

In *T. solium*, Ts24GST is abundant in the scolex and is associated with homeostasis processes related to detoxification [22]. Moreover, *E. granulosus* sigma‐class EgGST2 is associated with drug resistance and modulation of the host immune response [26]. In other helminths, such as *Fasciola hepatica*, sigma‐class GST has been identified in extracts of eggs and yolk cells and is associated with embryogenesis and reproduction. rFhGSTS1 can also synthesize prostaglandin D_2_ (PGD_2_) and prostaglandin E_2_ (PGE_2_) during infection to manipulate the host immune response [28].

The aims of this study were the structural characterization of the 24‐kDa glutathione transferase (*Ts24gst*) gene of *T. solium* and biochemical characterization of its protein product (Ts24GST). We identified the key motifs in the promoter for its expression, the splicing sites, and the sequence of the encoded mature protein. Moreover, we generated a theoretical 3D model and a recombinant enzyme to study the interactions with its substrates, its kinetic properties, and its stability at different temperatures and pH.

## Materials and methods

### Ethics statement

The institutional ethics committee approved all animal protocols (permission no. 2022‐435), which were carried out in strict accordance with the Official Mexican Norm for the Production, Care, and Use of Laboratory Animals (NOM‐062‐ZOO‐1999) and the Guide for the Care and Use of Laboratory Animals of the National Institutes of Health, USA.

### Gene analysis

We used the previously reported *Ts24gst* cDNA sequence as a probe [22] to identify the *Ts24gst* in the *T. solium* genome project (https://parasite.wormbase.org/Taenia_solium_prjna170813/Info/Index/) [29]. To confirm the sequence, we amplified the gene by PCR using specific oligonucleotides (Ts24GSTF: 5′‐GATCCTGCATACAATATTAAATC‐3′ and Ts24GSTR: 5′‐CATTAGATGATGTAGTCCATGG‐3′) and *T. solium* gDNA (PCR program: 94 °C for 1 min, 30 cycles at 94 °C for 30 s, 58 °C for 30 s, 72 °C for 2 min, and a final extension at 72 °C for 10 min). The PCR product was cloned into the pCRII vector (Invitrogen by Thermo Fisher Scientific, Waltham, MA, USA) and sequenced with an automated DNA sequencer (ABI Prism model 373; Applied Biosystems, Waltham, MA, USA). pcgene (IntelliGenetics, Inc., Atlanta, GA, USA) and clustal omega (www.clustal.org/omega) were used to analyze nucleotide sequences and multiple alignments. Finally, the AliBaba 2.1 server (http://gene‐regulation.com/pub/programs/alibaba2/) was used for the proximal and core promoter element analyses.

### Relative expression analysis

For qPCR, 3 μg of total RNA from larval and adult stages of *T. solium* were used for reverse transcription to cDNA using SMART Scribe Reverse Transcriptase (Clontech Laboratories, Inc., Palo Alto, CA, USA) according to the manufacturer's instructions. Next, 100 ng of cDNA were used in a final reaction volume of 10 μL per reaction. We used the designed primers GSTS‐X1 (5′‐CATATGGATTTACAACTTAAACAGG‐3′) and GSTS‐X2 (5′‐TTAGAAATCGGTAGCTGGGC‐3′) to amplify *Ts24gst*. *T. solium* Cu/ZnSOD amplification was done with the primers SOD‐6 (5′‐AAGCACGGCTTTCACGTCC‐3′) and SOD‐2 (5′‐ACGACCCCCAGCGTTGCC‐3′).

The reactions were performed using PowerUp SYBR Green Master Mix in a Step One Real‐Time PCR System (Applied Biosystems). The PCR scheme involved 95 °C for 10 min and then 40 cycles of 95 °C for 15 s, 60 °C for 1 min, and 72 °C for 30 s. The mRNA levels of *Ts24gst* were normalized to Cu/ZnSOD. The relative amounts of mRNA were calculated using the comparative CT method, and a *t*‐test was used for statistical analysis.

### Homology model of Ts24GST

A homology model based on a trained neural network for the Ts24GST was obtained using AlphaFold using and MMseqs2 in ColabFold (https://colab.research.google.com/github/sokrypton/ColabFold/blob/main/AlphaFold2.ipynb) with the default options. The best 3D *in‐silico* model was chosen based on the quality parameters of the model and MSA coverage. The structural model was refined by molecular dynamics simulations in explicit water using the gromacs package 2022.4 [30] in conjunction with the Amber force field [31] and the TIP4ε model to represent the water [32].

Energy minimization was performed, followed by equilibration in an NVT ensemble and then in an NPT ensemble. A production run was then performed for 100 ns. The quality of the obtained model was analyzed with psica [33], verify3d [34, 35] and errat software [36]. Favorable regions were identified by the Ramachandran plot obtained from procheck [37]. The theoretical molecular weight and isoelectric point (pI) were calculated with the Expasy server (web.expasy.org/compute_pi).

### Preparation of Ts24GST and ligand structures

Based on the minimized model, a search for potential binding sites was carried out using the DoGSiteScorer server and verified with the CASTp server and the SiteFinder function implemented in Molecular Operating Environment (MOE) software [38]. The G, L, and H‐sites presented drug scores of 0.82, 0.81, and 0.60, respectively, with a geometric center at 25.92 (*x*), 40.45 (*y*), and 34.90 (*z*). This center was chosen to perform a molecular docking. The coordinates of the rTs24GST, GSH, and 1‐chloro‐2,4‐dinitrobenzene (CDNB) structures were prepared using chimera 1.17.3 [39]. Gasteiger charges were added to the ligand, and AM1‐BCC was used for the receptor. For calculations, only polar hydrogens were added to all molecules.

### Molecular docking simulation

The GSH coordinates were downloaded (rcsb.org/ligand) and geometrically optimized using the OPLS‐AA force field method and MOE. Molecular docking was also performed using the rTs24GST model, and autodock vina 1.1.2 was implemented via chimera with 1 Å of spacing between the grid points. The docking results were verified by MOE. The grid box was centered according to the geometric center found by DoGSiteScorer.

### Bacterial expression and protein purification

The *Ts24gst* cDNA coding region was cloned into a pET22b expression vector (Novagen, Madison, WI, USA) and transformed in *Escherichia coli* BL21 (DE3) (Invitrogen, Carlsbad, CA, USA). As previously reported, PCR‐selected positive clones were sequenced and expressed at 37 °C with 0.3 mm isopropyl β‐d‐1‐thiogalactopyranoside (IPTG) [39]. Bacteria were harvested by centrifugation at 6000 ***g*** for 25 min at 4 °C, washed in 30 mL of 20 mm Tris–HCl at pH 7.4, and centrifuged again. The bacterial pellet was resuspended in the same buffer and lysed by sonication on ice in the presence of the “cOmpleteTM” protease inhibitor (Sigma‐Aldrich, St. Louis, MO, USA).

The rTs24GST was purified using 10 mL column UNOsphere cationic exchange resin (BioRad, Hercules, CA, USA) according to the manufacturer's instructions at a linear flow rate of 120 cm·h^−1^. The proteins bound to the column were eluted with a linear gradient of NaCl (0–1 m) in 20 mm Tris–HCl at pH 7.4 and collected in fractions of 2.0 mL. The fractions were analyzed using SDS/PAGE to identify those containing rTs24GST, which were pooled and concentrated in a centrifugal filter unit with molecular‐weight cutoff of 10 kDa to a concentration of 5.0 mg·mL^−1^.

A second purification step was performed by molecular exclusion chromatography, in which 10 mg of rTs24GST obtained in the first step was loaded in a Superdex 75 column (GE Healthcare, Chicago, IL, USA). The sample was eluted with 120 mL of 20 mm Tris–HCl at pH 7.4 and 150 mm NaCl with a flow of 1 mL·min^−1^. Fractions containing rTs24GST were dialyzed against 100 volumes of 20 mm Tris–HCl at pH 7.4 and concentrated to 5.0 mg·mL^−1^ for kinetics assays.

The column was calibrated with proteins of 78 kDa (maltose binding protein from *E. coli* with HilD fusion protein from *Salmonella enterica* serovar *Typhimurium*), 50 kDa (laccase from *Thermus thermophilus*), and 27 kDa (green fluorescence protein from *Aequorea victoria*). The protein concentration in all steps was determined by the Bradford method [40] and OD_260/280_ measurements in a nano‐drop spectrophotometer (Model ND‐2000; Thermo Fisher Scientific, Inc.). The protein's purity was verified by 12% SDS/PAGE on Coomassie blue‐stained gels. MALDI‐TOF and electrospray ionization mass spectrometry were performed in the Unidad de Proteómica of the Instituto de Biotecnología, UNAM.

### Antibody production

Two New Zealand rabbits (12 weeks of age) were immunized subcutaneously with 50 μg of rTs24GST plus 25 μg of Quil‐A adjuvant (InvivoGen, San Diego, CA, USA) in 0.5 mL of sterile saline solution four times at 2‐week intervals. Rabbits were anesthetized with barbiturates according to international anesthesia guidelines, and blood was collected by heart puncture at 60 days post‐immunization. The blood was refrigerated for 2 h at 4 °C and centrifuged at 5000 ***g*** for 15 min to obtain the serum.

### Western blot assay

*Escherichia coli* BL21 (DE3) crude extract, commercial alpha, mu, sigma, and omega recombinant GSTs from *Homo sapiens* (Oxford Biomedical Research, Oxford, UK), and rTs26GST (alpha‐mu), rTs25GST (mu), and rTs24GST (sigma) classes were separated by 12% SDS/PAGE in reduced conditions and then transferred to nitrocellulose membranes (BioRad) at 100 V for 1 h in cold conditions. Membranes were washed with PBS and incubated with anti‐rTs24GST serum (dilution 1 : 500) for 1 h at room temperature as the first antibody. The polyclonal second antibody (Jackson ImmunoResearch, West Grove, PA, USA) was an anti‐rabbit IgG coupled to peroxidase at a dilution of 1 : 2000 for 1 h at room temperature. Antibodies bound to the membrane were developed using a diaminobenzene solution (5 μg·mL^−1^) and H_2_O_2_ as previously reported [41].

### Kinetics of rTs24GST

Glutathione transferase activity was measured using a GSH conjugation substrate assay with CDNB [42]. The assays were performed in 1.0 mL of cells with 20 μg of enzyme in 1 mL of 50 mm Tris–HCl at pH 7.4 with 1.0 mm GSH. Each kinetic test was initiated with the addition of different volumes of 0.50 mm CDNB in pure DMSO. Then, absorbances were measured at 340 nm for 3.0 min at 25 °C in a Carry UV–Vis spectrophotometer (Agilent Technologies, Inc., Santa Clara, CA, USA), which was equipped with a Peltier system for temperature control. The specific activity was calculated in micromoles of conjugated product per minute per milligram of protein.

To assess the residual activity at different temperatures, 1.0 mg of rTs24GST was incubated at various temperatures in the range of 25–50 °C using a thermoblock (Eppendorf ThermoMixer® C, Enfield, CT, USA). The enzyme was maintained at each temperature for 24 h, and then its activity was measured as before. To evaluate the pH stability of rTs24GST in media at different pH, 500 μg of enzyme were dialyzed for 12 h at pH values ranging from 4.0 to 11.0 using the Britton and Robinson buffer system with 1‐unit difference between each sample. Experiments were conducted in triplicate. All statistical data were analyzed using prism 6.0 for Windows (www.graphpad.com). Statistically significant differences were determined using *P*‐values of less than 0.05 and a one‐way analysis of variance (ANOVA) and comparisons of data to the highest relative activity point.

### Circular dichroism

Far‐UV circular dichroism (CD) spectra were obtained with a JASCO J‐715 spectropolarimeter (Jasco Inc., Easton, MD, USA) equipped with a PTC‐348WI Peltier cell holder for temperature control. rTs24GST solutions of 0.10 mg·mL^−1^ in 20 mm Tris–HCl at pH 7.4 were used in a 0.10‐cm cell for each test related to temperature. The rTs24GST CD spectra were obtained in a temperature range of 20–60 °C with intervals of 5 °C between each measurement and equilibration using fresh protein solution at each temperature. The background signal of the buffer was determined and subtracted.

For pH characterization, the Britton‐Robinson buffer system was also used at pH between 4.0 and 11.0. The background signal was determined using 400 μL of Britton‐Robinson buffer at each pH value for each experiment. Finally, the background signal was used for subtracting the background noise as a control for the experiment. Each sample was measured in the range of 200–250 nm.

## Results

### Genomic analysis of *Ts24gst* and mRNA relative expression

**Fig. 1:**
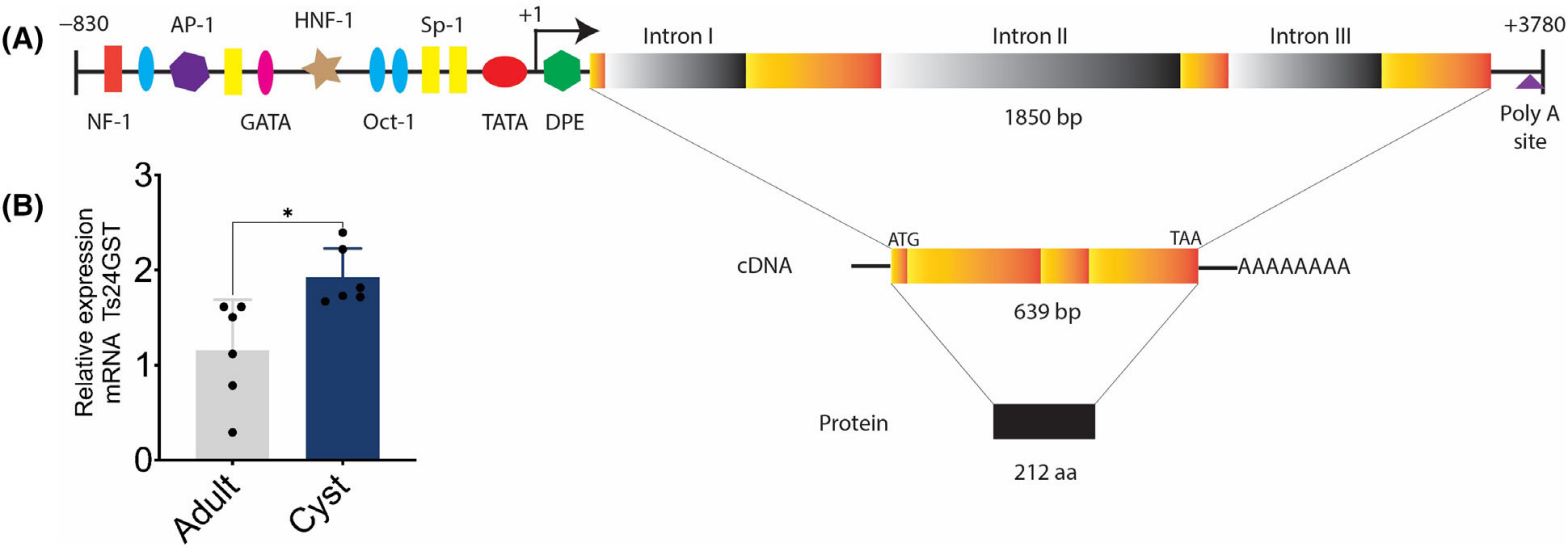
Gene structure of *Ts24gst* and relative expression. (A) The putative binding sites for transcription factors identified in the proximal promoter region: NF‐1 (red bar), Oct‐1 (blue oval), AP‐1 (purple hexagon), Sp‐1 (yellow bar), GATA (pink oval), HNF‐1 (brown star), TATA‐box (red oval), TSS (A_+1_, black arrow), and DPE (green hexagon). The coding region spans 1850 bp and presents four exons (orange bars) interrupted by three introns (gray bars). A polyadenylation signal (purple triangle) was identified in the 3′UTR. The cDNA spans 639 bp and encodes a polypeptide of 212 amino acid residues. (B) mRNA relative expression measured by qPCR for Ts24gst in cyst and adult stage (*n* = 6, *P < 0.05, *t*‐test, plot expressed as means ± SEM).

*Ts24gst* was identified in the *T. solium* genome project (contig_01131, gene ID TsM_000670100, UniProt C0M0N5_TAESO). No other similar sequences were found in the genome, suggesting that it is a single‐copy gene. Its genomic sequence spans 2900 bp. The analysis of the core and proximal promoter of *Ts24gst* showed putative sites for transcription factors such as NF‐1, Oct‐1, AP‐1, GATA, HNF‐1, Sp‐1, and TBP, as well as a transcription start site (TSS, CCATACT) that includes an A + 1. Moreover, a DPE (GGCTGA) was also observed (Fig. 1A, Fig. S1).

In addition, we observed regulatory elements in the distal promoter, such as the core sequence (GCGTG) of xenobiotic response elements (XRE) and binding sites for aryl hydrocarbon receptor (AhR) and/or AhR nuclear translocator (ARNT) in the forward and reverse strand in between −7500 and −2500 bp (relative to TSS). An AP‐1 binding site was also observed at approximately −680 bp. The coding sequence spanned 1850 bp and it was composed of four exons (exon I: 33 bp, exon II: 282 bp, exon III: 99 bp, and exon IV: 225 bp), which were split by three introns (intron I: 290 bp, intron II: 616 bp, and intron III: 316 bp). The intron/exon junction presented the NGT‐AGN donor‐acceptor sites needed for splicing.

A classic polyadenylation sequence (AAUAAA) was also observed at 3′UTR (Fig. 1A). *Ts24gst* produced a transcript of 639 bp containing start (ATG) and stop (TAA) codons and encoded a protein of 212 amino acids (Ts24GST) with a predicted molecular weight of 24.29 kDa and a theoretical pI of 9.22. We performed qPCR with cDNA from *T. solium* cysticerci and adults to determine the mRNA expression. The results showed a significant difference between the two stages with a relative expression of 1.15 AU (arbitrary units) in the adult and 1.92 AU in the cysticercus (*P* = 0.012 in a *t*‐test; Fig. 1B).

### Ts24GST protein analysis

**Fig. 2:**
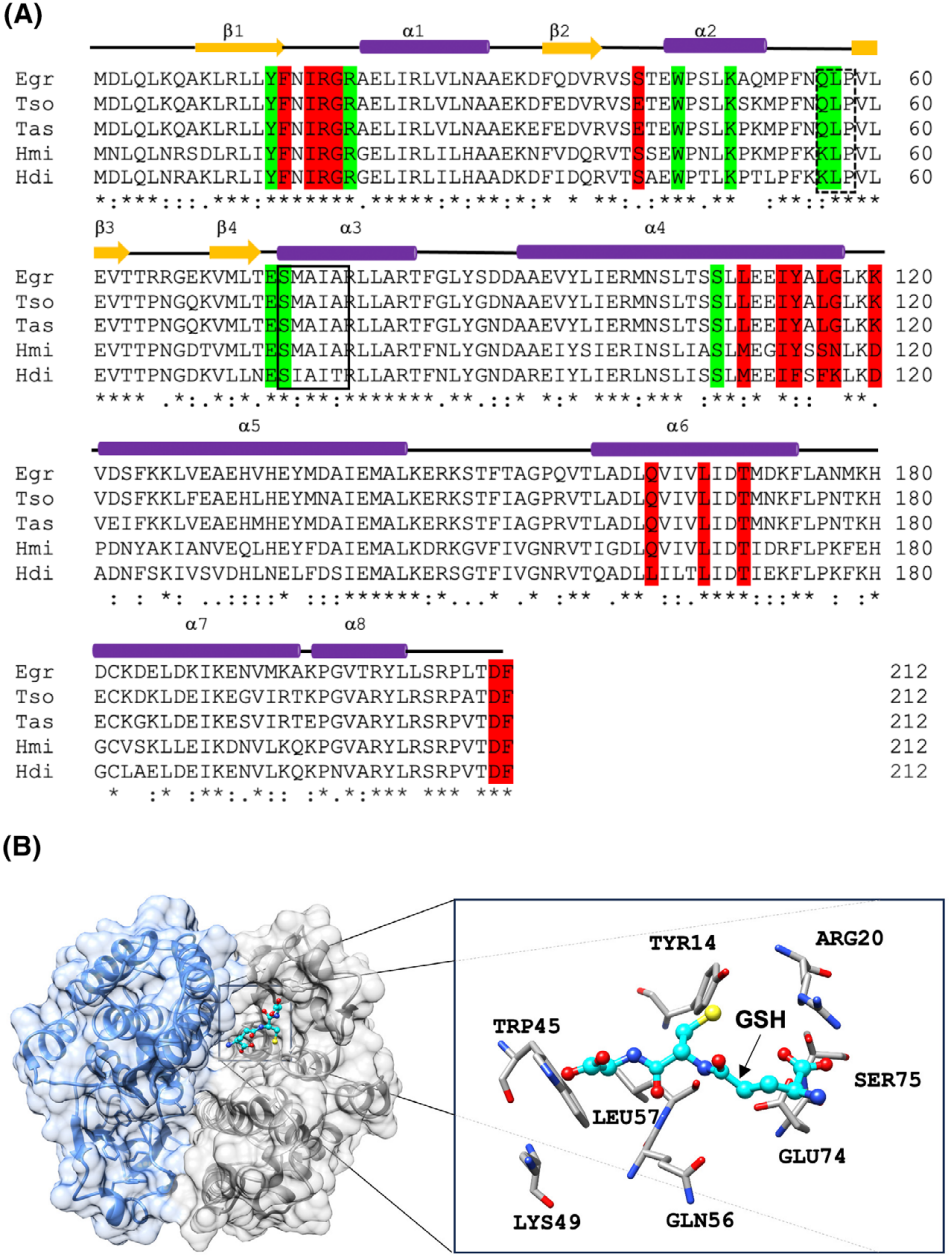
(A) Alignment of the primary sequence of Ts24GST with sigma‐class glutathione transferases from cestodes. Egr, *Echinococcus granulosus* ADQ89757.1; Hdi, *Hymenolepis diminuta* VUZ44991.1; Hmi, *Hymenolepis microstoma* CDS28394.1; Tas, *Taenia asiatica* VDK34811.1; Tso, *Taenia solium* ACN88552.1. The secondary structure is shown above the primary sequence. GSH and prostaglandin D_2_ binding sites are highlighted in green and red. The consensus SNAIL/TRAIL and cis‐Pro loop QVP/NLP motifs are shown in continuous and dashed boxes. Symbols indicate conserved (*) and homologs (:) residues. (B) 3D alpha‐fold model predicted for the dimeric state of Ts24GST (blue and gray). The box shows a magnification of the interaction between residues from the G‐site and the GSH (Cyan backbone).

The MALDI‐TOF spectrum shows a single symmetric peak with *m/z* 24.31‐kDa corresponding to common ion [M + Na]+(Fig. S2A). Therefore, the estimated molecular mass for rTs24GST is 24.29‐kDa, which fits well with the value obtained by primary sequence. Furthermore, electrospray ionization mass spectrometry analysis of the digested protein confirms the identity of rTs24GST (Fig. S2B). Its primary structure has 94% identity with *Taenia asiatica* sigma GST (VDK34811.1), 87% identity with *E. granulosus* sigma GST (ADQ89757.1), 67% identity with *Hymenolepis microstoma* sigma GST (CDS28394.1), and 66% identity with *Hymenolepis diminuta* sigma GST (VUZ44991.1) (Fig. 2A). The primary sequences were compared with another sigma‐class GST named hematopoietic PGD synthase (H‐PGDS, glutathione‐dependent) and the lipocalin‐type PGD synthase (L‐PGDS, glutathione‐independent) from *H. sapiens*, *Mus musculus*, and *Rattus norvegicus* with cestodes. The results showed that Ts24GST had around 27% identity with H‐PGDS and only 17.4% identity with L‐PGDS from the same species (Table S1).

The *in‐silico* 3D model of Ts24GST showed a typical GST dimer, showing that each monomer contains a small N‐terminal domain (residues 1–85) and a larger C‐terminal domain (residues 86–212). The N‐terminal domain is composed of 3 α‐helices (α1: residues E22 – A30; α2: residues W45 – K51; α3: residues S75 – T85) and 4 β‐sheets (β1: residues K9 – Y14; β2: residues E36 – R39; β3: residues V59 – T63 and β4: residues V70 – T73), which form the classic βαβαββα conformation presented in a thioredoxin‐like domain. Furthermore, a cis‐Pro loop QVP/NLP (residues 56–58) and a SNAIL/TRAIL motif (residues 75–79) were also presented in this domain. The C‐terminal domain is composed of 5 α‐helices (α4: residues 93–117; α5: residues 122–145; α6: residues 161–177; α7: residues 181–196 and α8: residues 198–205).

**Fig. 3:**
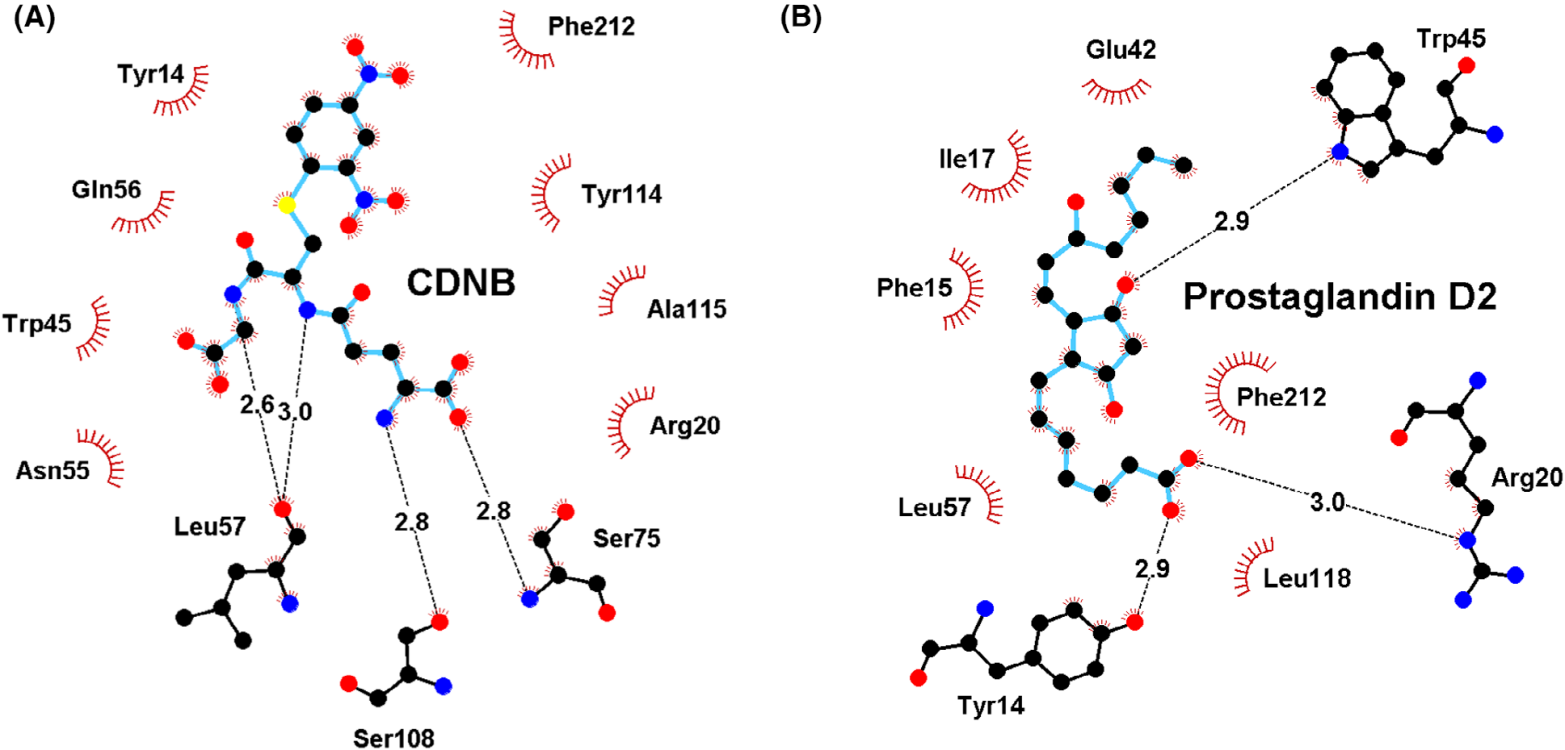
Predicted interaction of Ts24GST with two substrates in H‐site. (A) CDNB and (B) prostaglandin D_2_. The substrate backbone is shown in cyan, dashed black lines show hydrogen bonds, and red lines show non‐ligand residues involved in hydrophobic contacts.

The 3D *in‐silico* model also showed the interface of the dimer formed and a pocket that allows the entrance of the GSH to the G‐site. The molecular docking assays of the model revealed the conservation of amino acid residues involved in the GSH bound to the G‐site of a typical GST: Y14 (catalytic amino acid), R20, W45, K49, Q56, L57, E74, S75, and S108 (Fig. 2B). We also identified conserved amino acids that have been reported in the literature to bind CDNB, such as Y14, F15, R20, W45, F54, N55, Q56, P58, L57, T73, E74, S75, L105, S108, L110, I113, A115, Y114, A115, L118, K119, Y136, and F212 at the H‐site (Fig. 3A, Fig. S3). Interestingly, the comparison of *Onchocerca volvulus* GST1 (OvGST1) and the Ts24GST 3D *in‐silico* model revealed shared amino acids that conform to the prostaglandin D_2_ binding site, such as Y14, F15, I17, R18, G19, R20, E42, W45, L57, L110, I113, Y114, L116, G117, L118, K120, Q163, L167, T170, D211, and F212 (Fig. 3B) [43].

### Expression and purification of rTs24GST

**Fig. 4:**
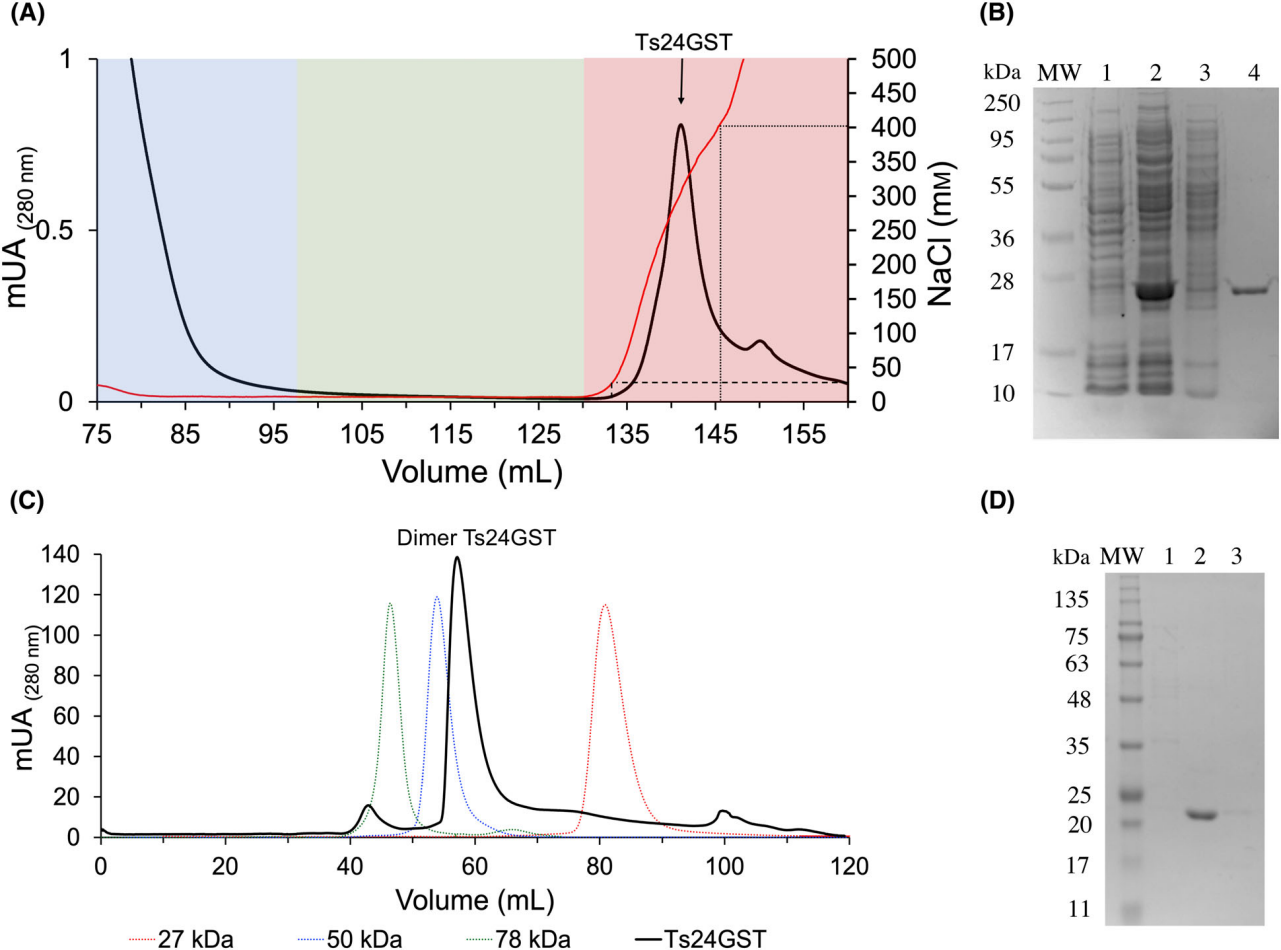
rTs24GST purification. (A) Cation exchange chromatogram. Non‐binding protein from crude extract of induced bacteria expressing rTs24GST (blue zone), resin wash (green zone), and elution of rTs24GST (red zone). (B) SDS/PAGE: molecular weight markers (MW); crude extracts from: (1) non‐induced bacteria; (2) induced bacteria; (3) passed through the cationic exchange resin; (4) rTs24GST eluted by NaCl gradient. (C) Molecular exclusion chromatogram of Superdex 75 column. The column calibration was done with 78, 50, and 27‐kDa proteins (green, blue, and red), black peak corresponds to rTs24GST. (D) SDS/PAGE showing MW; lanes: (1) eluted proteins of high molecular weight, (2) pure rTs24GST monomer, and (3) low‐molecular‐weight proteins.

rTs24GST was purified in two consecutive steps using cationic exchange and molecular exclusion chromatography. The cationic exchange column chromatogram (Fig. 4A) showed a single peak of protein eluted at 30 mm of NaCl. SDS/PAGE (Fig. 4B) showed the expression pattern of bacteria before (lane 1) and after induction (lane 2), the no‐binding fraction (lane 3), and the eluted fraction composed of a band of around 25 kDa (lane 4).

The first purification step removed most of the bacterial protein, yielding rTs24GST with 85% purity. The second purification step by molecular exclusion chromatography (Fig. 4C) showed three peaks. Peaks 1 and 3 were smaller than peak 2, which eluted a protein of around 50 kDa. SDS/PAGE showed that peak 1 represents a high molecular weight bacterial protein, peak 3 represents no detectable proteins, and peak 2 represents a protein of around 24 kDa (rTs24GST) with purity above 95% and a specific activity of 10.52 U·mg^−1^ with CDNB (Fig. 4D). The protein purification process yielded 78 mg of rTs24GST per liter of culture (Table 1).

**Table 1:** Purification by two steps for rTs24GST using 1 L of bacteria culture.

Extraction steps	Protein content (mg·mL^−1^)	Volume (mL)	Total protein (mg)	Specific activity (U^a^·mg^−1^)	Total activity (U^a^)	Yield (%)	Purification (fold)
Induced crude extract	161.6	30.0	4848	0.21	1019.08	100	1
Cation exchange chromatography	6.0	14.5	87	9.80	852.60	83.66	46
Size exclusion chromatography	20.0	3.9	78	10.52	820.56	80.51	50

^a^ A unit of activity is the amount of enzyme catalyzing the formation of 1 mol·min^−1^ of the product under the conditions of the specific assay.

### Cross‐reaction between *T. solium* and human GST classes

**Fig. 5:**
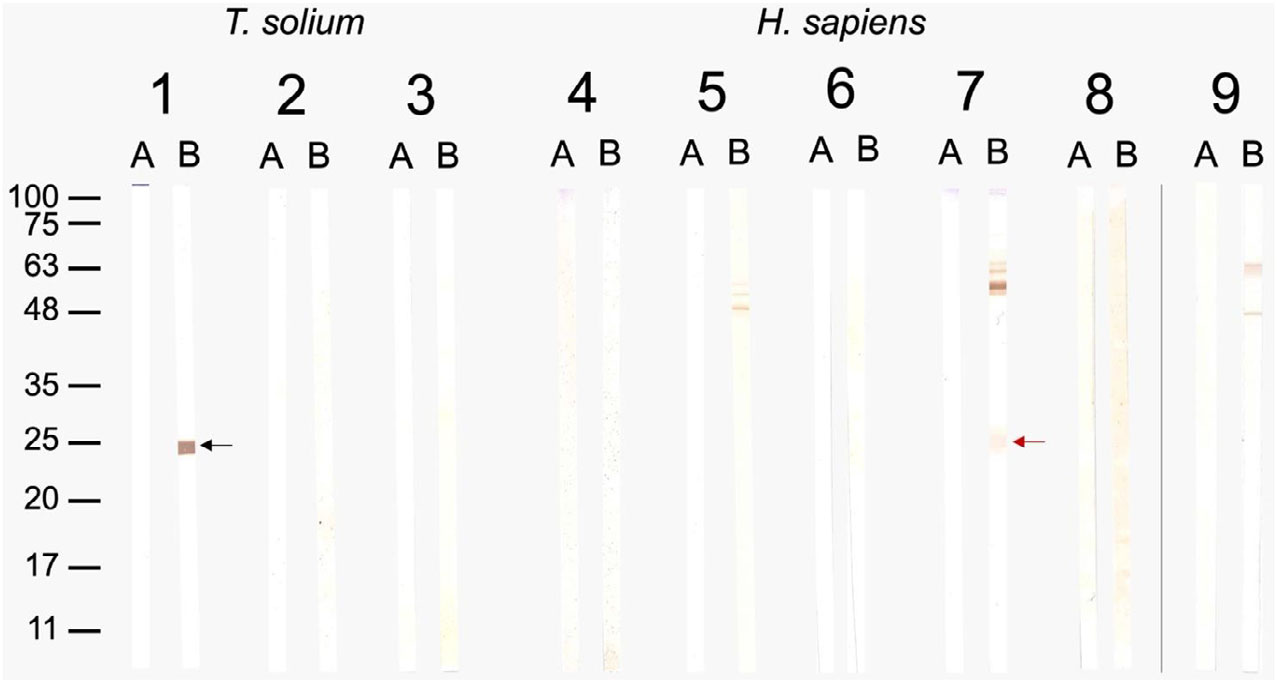
Western blot results of (A) preimmune serum (negative controls) and (B) anti‐Ts24GST serum against (1) rTs24GST (sigma, black arrow), (2) rTs25GST (mu) and (3) rTs26GST (alpha‐mu); (4) HsGST (alpha), (5) HsGST (mu), (6) HsGST (pi), (7) HsGST (sigma, red arrow), (8) HsGST‐omega classes and (9) *Escherichia coli* crude extract.

Rabbit anti‐rTs24GST serum was confronted with rTs24GST, rTs25GST, rTs26GST, commercial human GST classes (alpha, mu, pi, and sigma) and *E. coli* crude extract. The assay results demonstrated that anti‐rTs24GST strongly recognized rTs24GST (Fig. 5, line 1B), but not rTs25GST, rTs26GST (Fig. 5, lanes 2B, and 3B), or human alpha, mu, and omega GST classes. In contrast, the serum slightly recognized the sigma class of human GST (Fig. 5, line 7B). The bands with molecular weight higher than the GST recognized by the anti‐rTs24GST serum in the commercial human GST classes belonged to *E. coli*. Moreover, the rabbit anti‐rTs24GST serum did not recognize any band around the zone of 24 kDa in *E. coli* extract (Fig. 5, line 8B).

### Effect of pH and temperature on rTs24GST

**Fig. 6:**
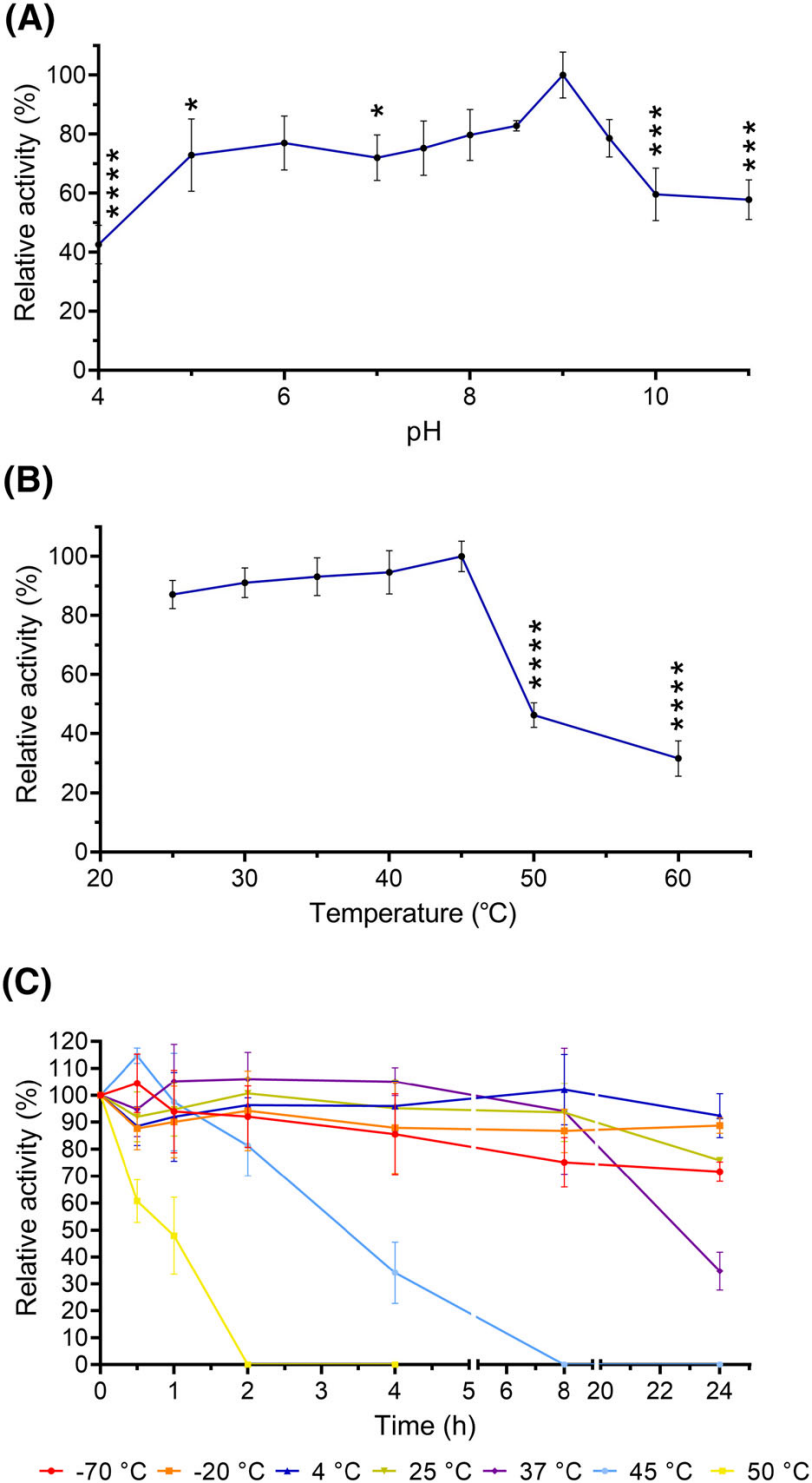
Determination of the relative activity of rTs24GST: (A) Plot of relative activity for pH 4–11 using constant temperature of 25 °C for 5 min. (B) Relative activity measured at 25–60 °C for 5 min. (C) Relative temperature measured for 0–24 h in a range of −70 °C to 50 °C. Plots express mean ± SD with *n* = 3. Panels A and B: *P < 0.05, **P < 0.01, ***P < 0.001, ****P < 0.0001, one‐way ANOVA.

In the pH analysis, rTs24GST showed 70% and 80% activity at a pH range of 5–8.5, and the highest activity occurred at pH 9 (100% activity). The activity declined by around 45% at pH 4 and at pH higher than 9.5 (Fig. 6A). In the temperature assays, rTs24GST exhibited a maximum activity at 45 °C. It maintained around 90% of its activity in the temperature range of 25–40 °C, while its in pH decreased to 40% and 20% of at 50 °C and 60 °C, respectively (Fig. 6B).

Considering the effect of temperature on the activity of rTs24GST, we measured the residual activity following 24 h incubation in a temperature range of −70 °C to 50 °C. Incubation at 50 °C for 0.5, 1, and 2 h caused the enzyme to lose 40%, 55%, and 100% of its activity, respectively (Fig. 6C, yellow line). At 45 °C, it showed an increase in activity of 15% with 0.5 h of incubation, but the activity gradually decreased to 95%, 80%, 35%, and 0% at 1, 2, 4, and 8 h of incubation, respectively (Fig. 6C, light blue line).

At 37 °C, the rTs24GST activity remained above 90% for 8 h and then decreased to 60% in 24 h (Fig. 6C, purple line). At temperatures between −20 and 4 °C, it maintained up to 80% activity for 24 h (Fig. 6C, orange and dark blue lines). When exposed to temperatures of 25 and −70 °C, rTs24GST maintain activity above 80% for 8 h, but its activity decreased by 70% and 75% after 24 h of incubation (Fig. 6C, olive green and red lines).

### Circular dichroism

**Fig. 7:**
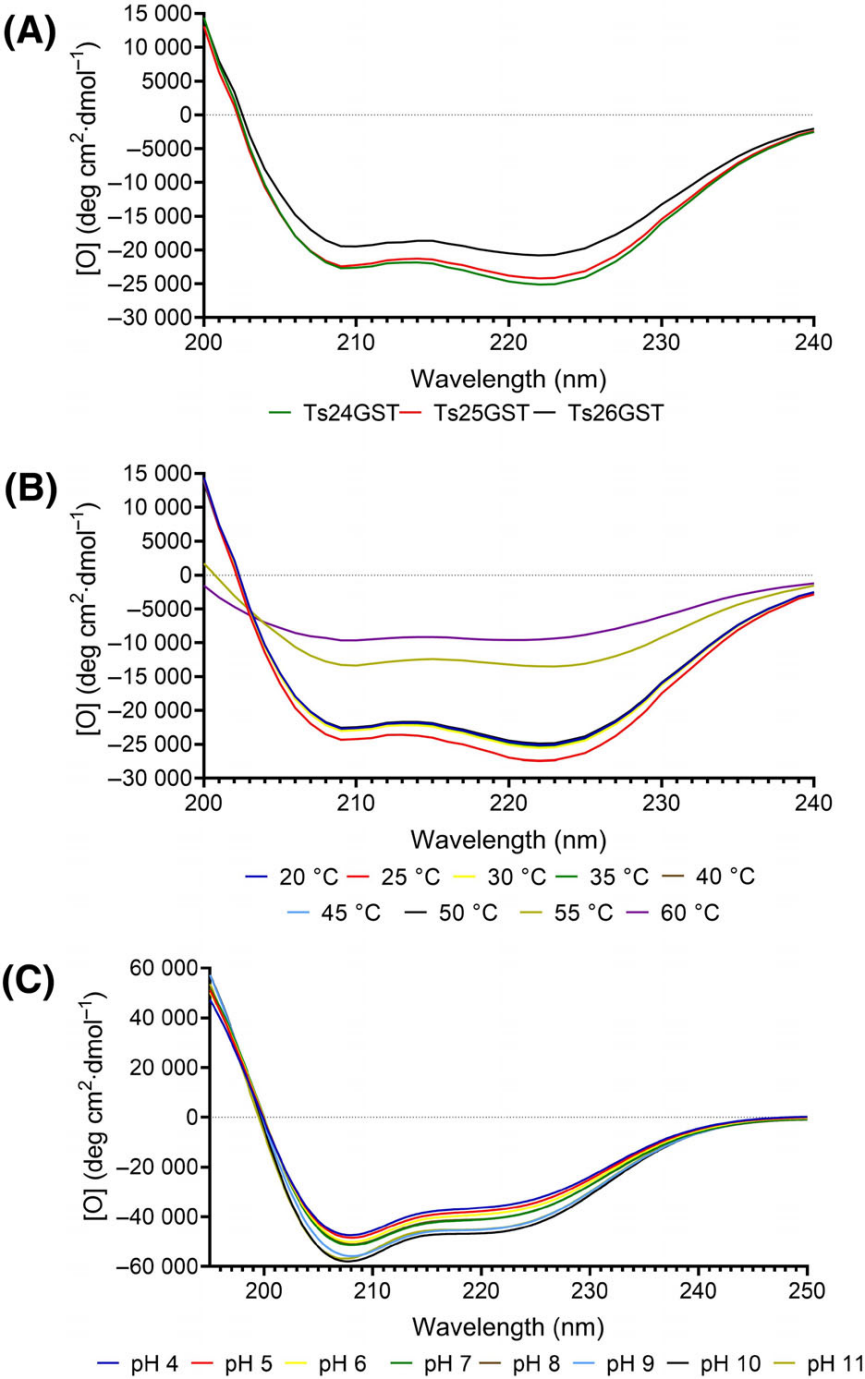
Circular dichroism spectra. (A) Comparison of structures from three recombinant enzymes of *Taenia solium* (Ts24GST, Ts25GST, and Ts26GST) measured at 25 °C. (B) Structural changes of rTs24GST incubated for 5 min between 20 °C and 60 °C. (C) Behavior of rTs24GST at pH 4.0–11.0.

The far‐UV CD spectra of the three cGST from *T. solium* (rTs24GST, rTs25GST, and rTs26GST) at 25 °C were very similar, and those from the first two GST were almost superimposable (Fig. 7A), despite sharing a very low sequence identity (rTs24GST sequence identity of 24.3% with rTs25GST and 22.9% with rTs26GST). These spectra are typical of proteins with significant contents of α and β structures. The CD spectra of rTs24GST indicated that it retained its secondary structure in a temperature range of 20–50 °C after 5 min of incubation.

However, the spectra of rTs24GST incubated between 55 °C and 60 °C showed a drastic decrease in ellipticity associated with a loss of the regular secondary structures. The main changes were observed as a flattening of the curve between 205 and 225 nm (Fig. 7B). The structural changes correlated with the activity loss observed in the enzymatic kinetics at these temperatures (Fig. 6C). DC was also used to characterize the change in the secondary structure of rTs24GST in response to pH values changes ranging from 4.0 to 11.0 (Fig. 7C). Alterations within this pH range did not show minimal alterations in the secondary structure of the enzyme.

## Discussion

The presence of sigma‐class GST in adult and cysticercus *T. solium*, especially in the scolex, suggests a potential role in several physiological processes. Such processes include detoxification of endogenous‐exogenous compounds and the regulation of host immune response, as reported in other organisms [26, 44, 45]. We characterized *Ts24gst* to test this possibility. *Ts24gst* spans 2900 bp and comprises four exons and three introns. This gene structure is similar to other cestodes but different from sigma‐class GST genes in mammals (humans and mice). These genes span 41 and 28 kb, respectively, and contain six exons and five introns, but the splicing motifs are identical in both organisms.

In the proximal promoter gene, we found putative binding sites for several general transcription factors related to the formation of transcription preinitiation complex, which were all localized at appropriate distances to be functional [46, 47]. These putative sites have been reported previously in *T. solium* genes [48, 49, 50, 51]. In addition, the presence of regulator elements such as XRE, AhR, ARNT, and AP‐1 were identified in the distal promoter of *Ts24gst*, which were are also found in promoters of GST genes in *E. granulosus*, as well as other GST and detoxifying enzyme genes [26, 52, 53].

Interestingly, we found no Nrf2 sites, which are present in GST genes that have a main function of detoxification. The qPCR assays showed differential expression of *Ts24gst*. Thus, in cysticerci, we observed an increase of 1.66‐fold compared to the adult stage. These data are comparable with the findings of Li *et al*. and other expressions of sigma‐class GST genes in parasites [17, 54, 55].

The gene architecture, splicing‐site conservation, distal and proximal elements found in the promoter, and similar molecular weight of the products that they encode suggest that *Ts24gst* is transcribed like mammalian GST genes are. *Ts24gst* also seems to be a housekeeping gene that can be induced by xenobiotics and drugs, as has been observed in other GST genes in different organisms [26, 52]. rTs24GST does not bind to a GSH Sepharose‐4B column, so its properties such as its predicted pI and molecular weight allow for the design of methods for its purification in two steps. The use of both cation exchange chromatography and molecular filtration chromatography allowed us to obtain a high yield of rTs24GST with purity close to 97%. The rTs24GST presented similar biochemical properties to previously reported properties of TsMαGST [22].

The Ts24GST *in‐silico* 3D model showed a structure consisting of an N‐terminal α/β domain (βαβαββα), in which strand 3 is antiparallel to the other strands. The C‐terminal domain contains five α‐helices and a long coil after the final helix. Most of the residues in the N‐terminal domain are mainly responsible for GSH binding. In the C‐terminal domain (H‐site), we found putative residues with a potential binding substrate site, like CDNB or PGD_2_ [56].

The alignments comparing Ts24GST with sigma GST classes denominated H‐PDGS showed high identity, which suggests that Ts24GST could have prostaglandin D_2_ synthase activity. In contrast, with our 3D model, it was impossible to identify the residues related to the ligandin site. Therefore, more structural and biochemical studies must be implemented to determine the capacity of putative rTs24GST to function as a molecular transporter [20]. Furthermore, this 3D model of Ts24GST could help to propose potential specific inhibitors.

Notably, the anti‐rTs24GST serum showed a slight cross‐reaction with only the human sigma GST class, but not with any other GST classes from *T. solium* and humans. This suggests that sigma GST classes share epitopes, which should be identified to develop vaccines or drugs to avoid autoimmunity and unspecific targets. In addition, other epitopes recognized by antibodies in serum anti‐rTs24GST could be used as targets to inhibit the enzyme or develop vaccines. Specific monoclonal antibodies against Ts24GST epitopes could also be used to classify helminth GST.

The induction of the *EgGST2* sigma gene by anthelminthic drugs suggests a role in drug resistance [26]. Moreover, rTs24GST has lower activity toward CDNB than rTs25GST and rTs26GST. Furthermore, according to Nguyen *et al*., it has null activity to substrates such as 1,2‐dichloro‐4‐nitrobenzene (DCNB), 4‐nitrobenzyl chloride (NBC), 2‐hydroxyl disulfide (HS), and ethacrynic acid (ECA). This suggests that like Ts25GST, Ts24GST has a small role concerning detoxification compared to Ts26GST [22, 23, 24, 41].

In contrast, Ts24GST showed putative prostaglandin D_2_ synthase activity, which suggests a role as an immunomodulator. PGD_2_ inhibits the NF‐ĸB signaling pathway, which induces a proinflammatory response [57, 58]. In addition, *Ts24gst* could be induced by oxidant stimulus of infections and increased synthesis of PGD_2_ [59, 60, 61], which binds to D‐prostanoid (PD) and chemoattractant‐homologous molecules on Th2 cell (CRTH) receptors, leading to suppression of the Th1 response. This could contribute to establishing the Th2 response, which favors the persistence of the parasite in the host and the absence of symptoms [62, 63].

Circular dichroism analysis showed that three *T. solium* GSTs (rTs24GST, rTS25GST, and rTs26GST) have the typical secondary structure of this family [23]. Physicochemical factors such as temperature and pH affect the tertiary structure and activity of helminth GST [64, 65]. We observed that rTs24GST maintains activity above 70% and 80% in wide ranges of pH and temperature, as in the cases of Ts25GST and Ts26GST [23].

In addition, thermal stability, pH, and temperature CD assays show that rTs24GST has a stable structure. This allows the cavity of G‐site to favor a p*K*_a_ of value of 8.45 for the thiol group, which promotes the formation of thiolate [66]. It could also have importance in the survival and establishment of *T. solium* eggs and adults, which live outside and within the intestine of humans, respectively, which vary in temperature between 0 °C and 50 °C and between pH 5 and 8 [67, 68, 69]. Finally, the *T. solium* GST system could be a drug target due to its participation in essential metabolic processes, such as the transport of molecules, signaling pathways, resistance, detoxification, and immunoregulation. More experiments are needed to clarify these questions.

## Conflict of interest

The authors declare no conflict of interest.

### Peer review

The peer review history for this article is available at https://www.webofscience.com/api/gateway/wos/peer‐review/10.1002/2211‐5463.13795.

## Author contributions

RM‐B, OR‐L, PG‐G, RF‐L, LJ, RAZ, ER‐P, and AL performed experiments and analyzed the data. RM‐B and AL designed the experiments. RM‐B, OR‐L, ER‐P, PG‐G, RAZ, and AL drafted the manuscript. All authors have read and approved the final manuscript.

## Supporting information


**Fig. S1.** Genomic structure of *Ts24gst*.

**Fig. S2.** Protein identification.

**Fig. S3.** Multiple sequence alignment for sigma‐class glutathione transferases.

**Table S1.** Identity matrix of an alignment between Ts24GST with Prostaglandin D synthases.

## Data Availability

All data supporting the findings of this study are available within the paper and its [Link], [Link], [Link], [Link]. Additional requests can be obtained from the corresponding author: landap@unam.mx.
