# Supplementary material for: Biochemical characterization and gene structure analysis of the 24‐kDa glutathione transferase sigma from Taenia solium
Source: FEBS Open Bio. 2024 Mar 21;14(5):726–39. doi: 10.1002/2211-5463.13795 (PMC11073501; doi:10.1002/2211-5463.13795)
Supplement: Supplementary file 1 — Fig. S1. Genomic structure of Ts24gst. [file FEB4-14-726-s002.pdf]

```
                                    XRE
-7546 ...//AAAATGGGTATCTAATGGCGTGCGAATGTTAGTTTAAAAAATACCTATGC//...
                                                           XRE
-7241 ...//TTTCTGTGCCCCATATACAGTCAGGCAGGTGTGTAATCGCCTCCACGCGT//...
                            XRE
-5986 ...//ACGTTTCTACACGCTCGCTCAAATTTCACCCTATATTACGCACCTGGTAG//...
                                                     XRE
-5626 ...//CTTTGTTTTTGATCTCCATGTATCGAATGAGCAGGCGTGAAAGGTACAGT//...
                                           XRE
-5266 ...//TCTTCTGTCCACCTTTGATAAGCCCACGCACGCATCGCCACCGTTCCCGA//...
                                    XRE
-4846 ...//GCTGATATCTGTTCTATGCGTGGGAGCAGTCGTCATAGGTGTGATAGTTG//...
                                         XRE
-3946 ...//CCACCGCAGATCTGTTACCGTAGCGTGGCTGGTTTTTTTTGTCACTTTTC//...
                        XRE
-2326 ...//ATTCATGCGTGACATTTGAACTTTGTGAAAAGGCAATTTAAAGAGTTTTG//...
                                             NF-1
 -831 GATCCTGCATACAATATTAAATCTGCGGAAATAAGGCCAACTTAAAGTGAAGTTGAACTC
 -771 TTCAACTGCCTTTAATCGTCGAAGAATAGAACAATTTCAGCACAAAGGAAGACGACCCGG
       Oct-1                               AP-1
 -711 ATTTACAAAACTGTTGAAAAAATGGTAGTACACTAAGTCATTTTTTTTCGAGAACTATTT
                Sp-1                                           GATA
 -651 GAAAAGTGACTGTGAAGTGGAGGAGATACCAACGGTTGCATCTTCTCGGCTTTTCATTTG
 -591 AGTTTACCTATCGGCTGTCGCCAGTTTCTCGTTCGCAATGTGCGTTAAAAACACTTTGAT
 -531 CTTCATCACTACATCTAGAACTAGGCTACGCGTCATAACTCCTTTTCTACTTTAACAATT
           HNF-1
 -471 AGCTATATTTGTTAATACTTTGAGTAATGGAAAAGATAGGATACTTGTTGGAATCTTCGT
       Oct-1
 -411 AATTACATTGTGCACAAAATTTAGGTGCGCCTCATATTTCCCGCACAAAATTGCGGCATT
                                 Oct-1
 -351 AGGACTGTCGGAAGAGTTTATGCGTAGCAAGAATTGCAATCGCACAGACGTGAATGTTTC
 -291 GCAGATGGGAAGTGTTTCGAGATTTTCCTGGTGTAAACGGTGGCTACGTACCGGTCCACA
 -231 GCTAGCAGTCATTGCACAGGTTGCGATGCAAGTTACATCACTTCAGTAAAACTGGAAGAA
                              Sp-1         Sp-1
 -171 TTGAGGTTATGAGCCAGTGCCGAAGCCCCTCTTATGAGGAAGGCGAACTAAAGGAAGGAC
                                 TATA-Box
 -111 AACACAATATCTTGTTTGTTCATCAATAAAAGAACGTGCCTCATTATTGCACCATTGCCA
        TATA-Box                                         Inr
  -51 CGTTTAAGCTGGCATGAATCATGAGGCAGGTACTTTGGCTCACACTGCGCCATACTCTGG
                                         DPE
   10 GTAATGTCTCGCATTCCAGGAGCTGGATTAGGCTTATTGGCTGAGCCAAGGTTACCAGCC
   70 CCTTGTTCTGCACTAAGTGAGTGTTGTGCCCTACATAGTGTGCACACACAAGAAACATGT
  130 ACCAGTTGCACCATCTTCGGGATAGATCAGCACCAGCGCCATGGATTTACAACTTAAACA
                                              M  D  L  Q  L  K  Q    7
  190 GGCCAAATTAAGGGTGAGGTCCCTGCACGATTCAATGTAGTCGCCATCTGATTTATCCTT
        A  K  L  R                                                  11
  250 ATACCTATCGTAACTTTAACGCGCTAATTACTGCCCTTGAGATGGCTTTTATCATCGACA
  310 TTCCCATCGTCACGAATAAATGTAACGTGAGGGATTATCCTCTGACCTGTTCCATAATCT
  370 ACGAACTTCGATAAGATCGCTAATTAAACTCATTTTGAGCTAATGTAGATTACCACTATC
  430 CAATGTTTAAGTATCTCTAATCTTTAAAATTTTTACCCCTATTTCTACATTAATTTTTTC
  490 CAGCTGCTTTACTTCAATATTCGTGGTCGTGCAGAGTTGATTCGACTGGTGCTGAATGCC
         L  L  Y  F  N  I  R  G  R  A  E  L  I  R  L  V  L  N  A    30
  550 GCAGAGAAGGACTTTGAGGATGTACGTGTAAGTGAAACTGAGTGGCCCTCACTGAAGTCC
       A  E  K  D  F  E  D  V  R  V  S  E  T  E  W  P  S  L  K  S    50
  610 AAAATGCCCTTCAACCAGCTGCCCGTGCTGGAGGTCACAACACCAAACGGCCAGAAAGTT
       K  M  P  F  N  Q  L  P  V  L  E  V  T  T  P  N  G  Q  K  V    70
  670 ATGCTCACGGAGAGTATGGCCATCGCCCGTCTGCTAGCGCGCACCTTCGGCCTCTACGGT
      M  L  T  E  S  M  A  I  A  R  L  L  A  R  T  F  G  L  Y  G    90
```

```
 730 GATAACGCCGCCGAAGTCTACCTTATTGAGCGAATGAACTCCCTTGTAAGTGAGTGCATT
      D  N  A  A  E  V  Y  L  I  E  R  M  N  S  L                  105
 790 GGCCCTCGTGTATGTTTCATAAAGCCCGCCAGTGAACTCAAAACATTCTGGATGGTAAGA
 850 CATAAGAATGACACCTTGTTTACAGTAAGAAAATAATTAGTTAGCATTTGGCCAGTCAGT
 910 TTAGACATAATTACAAAAACAAGCTGAATCTGTGATGCCCTAGCATTATGAGCACATTCT
 970 TTCTCATTCTGTTGTTGAAGATTCTCAATAAAACAGATTTCACACTGCGTAAATAATCTG
1030 GAATTTGGGAATTTAACACGACACAACACTTAAACGACATTAAAAGGGATAGTAAACAGT
1090 CTTACAATAGATTATGGTCAGCTAATTGGGAATTGACTCACCTTTGTCATGGTTTTCTCG
1150 CAAGGGGCAGGTAAATGGATACATCATAAATCTTGCAAAAACATCCAAAATAGCACAATC
1210 AAGCAGAAATTTTTCATGATAGAGTACCATCCTGCCGATTATAACATTTTTAGATTAATA
1270 AGTAACTTTGAGGCTTAAGAGTGTCGAATGACAGGCGAGGTGAGGTGATGAAACAGTACA
1310 ACATCAGCTAGGTACCCAACCACTACATCAACACGAATTACGCCAAGTTTCCGCTTTATA
1390 GACGAGTTCCCTCTTGGAGGAAATCTATGCCTTGGGCTTGAAGAAGGTCGACAGTTTTAA
       T  S  S  L  L  E  E  I  Y  A  L  G  L  K  K  V  D  S  F  K 125
1450 GAAATTGTTTGAAGCGGAGCACTTGCACGAGTACATGAATGTAAGTCCACCACCTCCTTT
       K  L  F  E  A  E  H  L  H  E  Y  M  N                        138
1510 CATCATTAGCGTCGCTGGCAGGCAGTACTCGTTAGTTCTCTTTAATTTATGCTTTATCAC
1590 AGTCAGCAACGCCCTTCTCATACTAGCAAATAATGACAACCCCCACTTGGCACAGAAGAT
1650 AAATAATGCGCCCTCCGAGACTGAATCTTGAGGGCCTAAACCCTTTTTGATTGACAGCGC
1710 CAAACAGCATTTTCGCAACTAAGCCAACCGCGATTTCGTATCACATAGCGGTACTCACAA
1790 GGCTACTTCACTAAACCTTTTAATACGTCCCACTTTCCCCGAAATTCTTTGTTAAGGCAA
                                                              A   139
1850 TTGAAATGGCTCTGAAAGAACGGAAGAGCACATTCATCGCAGGACCTCGGGTCACCTTAG
     I  E  M  A  L  K  E  R  K  S  T  F  I  A  G  P  R  V  T  L   159
1910 CCGACCTCCAAGTGATAGTTCTAATCGACACAATGAACAAATTTCTTCCGAACACAAAGC
     A  D  L  Q  V  I  V  L  I  D  T  M  N  K  F  L  P  N  T  K   179
1970 ACGAGTGCAAGGACAAATTGGACGAAATCAAGGAGGGTGTTATCAGGACAAAGCCTGGAG
     H  E  C  K  D  K  L  D  E  I  K  E  G  V  I  R  T  K  P  G   199
2030 TCGCCAGATACCTCCGTTCACGCCCAGCTACCGATTTCTAATGTTTGACCCTTACATTTC
     V  A  R  Y  L  R  S  R  P  A  T  D  F  -                     212
2090 TACCGCCATTGTTAGGACTCCCAGTTTCTATGGTATTTAACATGAAGTTAGTGCCACCAT
2150 TCTCGAACTTAATAAGTGAGCGGAAGGCAAGTAAAGTAAGACAGAAACAAATAATGTATA
2210 GGCTGTCAGCTTTAGAAATAAGTATAACATGCATGCTGAGTCCACATTTTTCACCCACAG
2270 CTGACTGCAGGATGCAAATGTGCAAAACAAAACTTCTTTCCCATTATTCGGCAGAAAAAA
2330 TGCGGGAAGACACGTCTTTGATAAGCCACGCCACACTGTTCAGAAGGTTTTCCCCCGTTA
2390 CAGCACTACAGCCGTAAATACTCCAGTGGTGTGTCGTAACATCCTTGAGACTGAGCGCCT
2410 CAGCTATTTCAGCAGGTTTTAATGCGCCTTGTAGATCCTACAGGACATAAGGGTAAGGAT
2470 CGGGGACAAGCCTTATTAGGCAAATGGTCTTCAAAGGCAAAAATATAAGTACAACAGAAA
2530 ACAAATTGATGACGAGACAAAAAGTAAAAGCGTATAGTAATAAGATACTCAAGTACTCAC
2590 CTGTTTATTTGCTAGTACGAGAAGAGTTGCTCCAACCAATCGCTCTTCCACTAGCAACTT
2650 GTTTAACTCCTCACGACAGTCATCTAACCGCAACCGATCGGAGCTGTCGACGACCCAAAT
2710 GAGAGCATCCGTAGTCTCGAAGTAATTACGCCAGTAAGAGCGTAAAGAATGTTGACCACC
2770 GACATCCCAGGCATTCAGCCTGTAGCCCATATACTCCAGAGTATGAATACTGAATCCCAG
2830 AGTAGGTGCGATTGTGGATATTTCCTCACCATTGAACTTTTTGACAATGGTTGTCTTGCC
2890 AGCATTATCCAATCCACTGCATAACAGAATGCAAGTAATAAAGCGCTGATGGAAGTGCAC
2950 TAAAGCAGTGTAACGTTGTAACGTATGTCACATTTCCACCATGGACTACATCATCTAATG
```

Supplementary figure 1. Genomic structure of *Ts24gst* (GenBank accession number: C0M0N5_TAESO). Putative transcription factors binding sites are written above their highlighted target sequence. Core xenobiotic response elements (XRE) are in cyan, TATA-box binding sites are in red, DPE site is in gray. Putative TSS is double underlined and identifies as Inr. Start (ATG) and stop (TAA) codons are in yellow. Donor and acceptor intron sequences (**GT**/../**AG**) are in bold black letters. Introns are underlined. The polyadenylation site is in magenta. Numbers to the left correspond to nucleotides, and numbers to the right correspond to amino acids.
