# Supplementary material for: Biochemical characterization and gene structure analysis of the 24‐kDa glutathione transferase sigma from Taenia solium
Source: FEBS Open Bio. 2024 Mar 21;14(5):726–39. doi: 10.1002/2211-5463.13795 (PMC11073501; doi:10.1002/2211-5463.13795)
Supplement: Supplementary file 2 — Fig. S2. Protein identification. [file FEB4-14-726-s001.pdf]

A

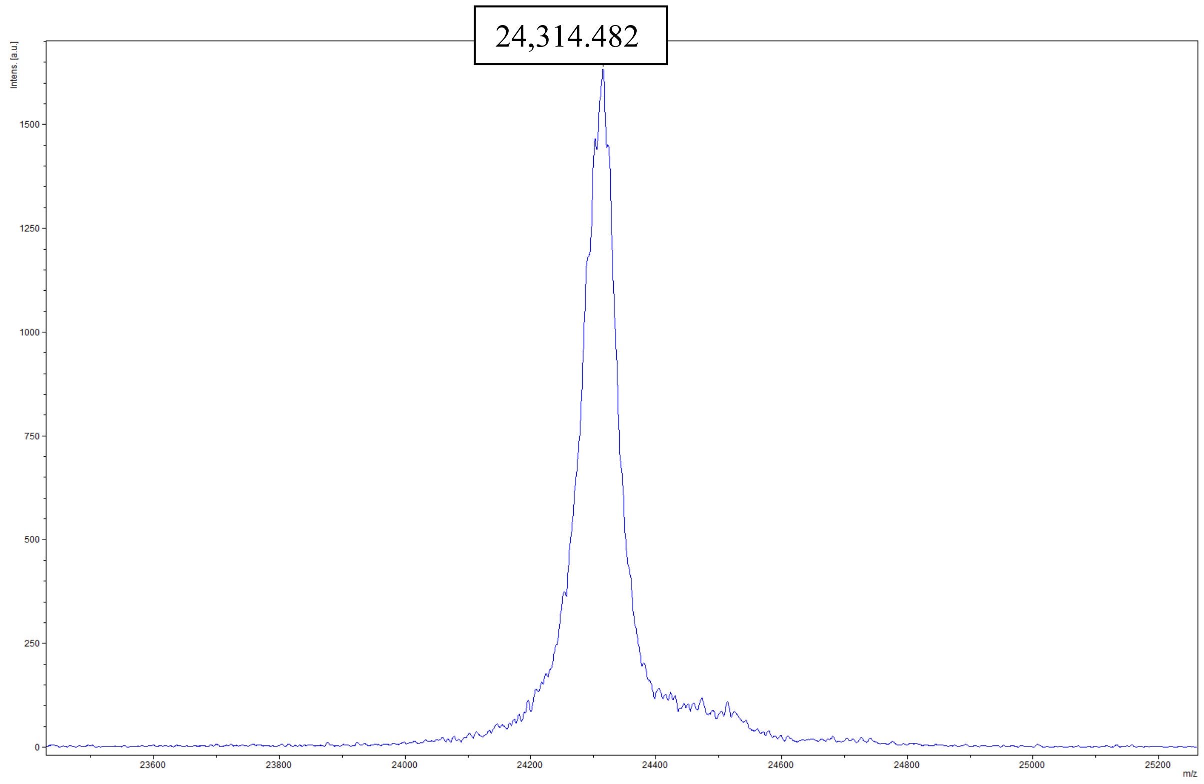

B

Sigma-type glutathione transferase OS=Taenia solium OX=6204 GN=GST-28 PE=2 SV=1

MDLQLKQAKLRLLYFNIRGRAELIRLVLNAAEKDFEDVRVSETEWPSLKSKMPFNQLPVL
EVTTPNGQKVMLTESMAIARLLARTFGLYGDNAAEVYLIERMNSLTSSLLEEIYALGLKK
VDSFKKLFEAEHLHEYMNAIEMALKERKSTFIAGPRVTLADLQVIVLIDTMNKFLPNTKH
ECKDKLDEIKEGVIRTKPGVARYLRSRPATDF

*Yellow: Amino acid sequence identified
*Green: Oxidated and deamidated amino acids
*No highlighted: No identified sequence

Protein Name	Protein Accession	Protein Identification Probability	Protein Percentage of Total Spectra	Exclusive Unique Peptide Count	Exclusive Unique Spectrum Count	Exclusive Spectrum Count	Percentage of Amino Acid Identified	Protein Molecular Weight (Da)
Sigma-type glutathione transferase	C0M0N5	100	24	24	66	1367	83%	24,293

Supplementary figure 2. Protein identification. **A)** The MALDI spectrum of rTs24GST (50 µg/mL in 10 mM Tris-HCl pH 7.4) was obtained in positive mode in a MALDI-TOF/TOF AutoFlex Bruker. The m/z value shown in the spectrum corresponds to the [Ts24GST + Na]+ = 24291.39 Da + 22.9898 Da = 24314.38 Da. **B)** The identification results of the purified rTs24GST by Electrospray Ionization Mass Spectrometry.
