# Supplementary material for: Biochemical characterization and gene structure analysis of the 24‐kDa glutathione transferase sigma from Taenia solium
Source: FEBS Open Bio. 2024 Mar 21;14(5):726–39. doi: 10.1002/2211-5463.13795 (PMC11073501; doi:10.1002/2211-5463.13795)
Supplement: Supplementary file 3 — Fig. S3. Multiple sequence alignment for sigma‐class glutathione transferases. [file FEB4-14-726-s003.pdf]

```
HsGSTS    ------MPNYKLTYFNMRGRAEIIRYIFAYLDIQYEDHRIEQADWPEIKSTLPFGKIPIL   54
MmGSTS    ------MPNYKLLYFNMRGRAEIIRYIFAYLDIKYEDHRIEQADWPKIKPTLPFGKIPVL   54
RnGSTS    ------MPNYKLLYFNMRGRAEIIRYIFAYLDIKYEDHRIEQADWPKIKPTLPFGKIPVL   54
Ts24GST   MDLQLKQAKLRLLYFNIRGRAELIRLVLNAAEKDFEDVRVSETEWPSLKSKMPFNQLPVL   60
                  : :* **::***** ** ::     .:** :.   :** :* .:**.::*:*

HsGSTS    EVDGL-----TLHQSLAIARYLTKNTDLAGNTEMEQCHVDAIVDTLDDF-MSCFPWAEKK  108
MmGSTS    EVEGL-----TIHQSLAIARYLTKNTDLAGKTALEQCQADAVVDTLDDF-MSLFPWAEKD  108
RnGSTS    EVEGL-----TLHQSLAIARYLTKNTDLAGKTELEQCQVDAVVDTLDDF-MSLFPWAEEN  108
Ts24GST   EVTTPNGQKVMLTESMAIARLLARTFGLYGDNAAEVYLIERMNSLTSSLLEEIYALGLKK  120
          :*           :*:**** *::   : *..  *    : : .  ..:  . :

HsGSTS    QDVKEQMFNELLTYNAPHLMQDLDTYL--GGREWLIGNSVTWADFYWEICSTTLLVFKPD  166
MmGSTS    QDLKERMFNELLTHQAPRLLKDLDTYL--GDKEWFIGNYVTWADFYWDICSTTLLVLKPG  166
RnGSTS    QDLKERTFNDLLTRQAPHLLKDLDTYL--GDKEWFIGNYVTWADFYWDICSTTLLVLKPD  166
Ts24GST   VDS---FKKLFEAEHLHEYMNAIEMALKERKSTFIAGPRVTLADLQVIVLIDTMNKFLPN  177
           .      : :   :    :: :.  *       . *  :* .*:        :    *.

HsGSTS    LLDN-HPRLVTLRKK-VQAIPAVANWIKRRPQTKL                          199
MmGSTS    LLDI-YPKLVSLRNK-VQAIPAISAWILKRPQTKL                          199
RnGSTS    LLGI-YPRLVSLRNK-VQAIPAISAWILKRPQTKL                          199
Ts24GST   TKHECKDKLDEIKEGVIRTKPGVARYLRSRPATDF                          212
                 :*  :::     :   :: ::  *  * :
```

Supplementary figure 3. Multiple sequence alignment for sigma class glutathione transferase from *Homo sapiens* (Hs); *Mus musculus* (Mm); and *Rattus norvegicus* (Rn). The amino acid involved in interaction with CDNB are highlighted in red. Symbols indicates residues conserved (*), and homologues (:). Numbers to the right correspond to amino acid residues.
