# Supplementary material for: Biochemical characterization and gene structure analysis of the 24‐kDa glutathione transferase sigma from Taenia solium
Source: FEBS Open Bio. 2024 Mar 21;14(5):726–39. doi: 10.1002/2211-5463.13795 (PMC11073501; doi:10.1002/2211-5463.13795)
Supplement: Supplementary file 4 — Table S1. Identity matrix of an alignment between Ts24GST with Prostaglandin D synthases. [file FEB4-14-726-s004.pdf]

Supplementary table 1. Identity matrix of an alignment between Ts24GST with Prostaglandin D synthases, lipocalin type (L-PGDS) and hematopoietic (H-PGDS) of mammals (Human: Hs; rat: Rn and mouse: Mm). The identity between them is highlighted in green, and the identity between Ts24GST against H-PGDS and L-PGD is highlighted in blue and yellow, respectively.

	Hs L-PGDS	Rn L-PGDS	Mm L-PGDS	Ts24GST	Hs H-PGDS	Mm H-PGDS	Rn H-PGDS
Hs L-PGDS	100	69.31	72.49	17.79	13.38	14.01	13.38
Rn L-PGDS	69.31	100	88.36	17.28	16.67	16.67	16.03
Mm L-PGDS	72.49	88.36	100	17.28	14.74	14.74	14.10
**Ts24GST**	**17.79**	**17.28**	**17.28**	**100**	**28.06**	**27.04**	**25.51**
Hs H-PGDS	13.38	16.67	14.74	28.06	100	80.40	81.41
Mm H-PGDS	14.01	16.67	14.74	27.04	80.40	100	93.97
Rn H-PGDS	13.38	16.03	14.10	25.51	81.41	93.97	100
